# Additions to the taxonomy of *Lagarobasidium* and *Xylodon* (Hymenochaetales, Basidiomycota)

**DOI:** 10.3897/mycokeys.41.28987

**Published:** 2018-10-23

**Authors:** Ilya Viner, Viacheslav Spirin, Lucie Zíbarová, Karl-Henrik Larsson

**Affiliations:** 1 Lomonosov State University, Faculty of Biology, Leninskie Gory 1/12, 119234 Moscow, Russia University of Helsinki Helsinki Finland; 2 Botany Unit (Mycology), Finnish Museum of Natural History, P.O. Box 7, FI-00014 University of Helsinki, Finland Lomonosov State University Moscow Russia; 3 Natural History Museum, University of Oslo, P.O. Box 1172, Blindern, 0318 Oslo, Norway University of Oslo Oslo Norway; 4 Resslova 26, Ústí nad Labem, CZ-400 01, Czech Republic Unaffiliated Ústí nad Labem Czech Republic

**Keywords:** Agaricomycetes, *
Hyphodontia
*, ITS, LSU, phylogeny

## Abstract

*Lagarobasidium* is a small genus of wood-decaying basidiomycetes in the order Hymenochaetales. Molecular phylogenetic analyses have either supported *Lagarobasidium* as a distinct taxon or indicated that it should be subsumed under *Xylodon*, a genus that covers the majority of species formerly placed in *Hyphodontia*. We used sequences from the ITS and nuclear LSU regions to infer the phylogenetic position of the type species *L.detriticum*. Analyses confirm *Lagarobasidium* as a synonym of *Xylodon*. Molecular and morphological information show that the traditional concept of *L.detriticum* covers at least two species, *Xylodondetriticus* from Europe and *X.pruinosus* with known distribution in Europe and North America. Three species currently placed in *Lagarobasidium* are transferred to *Xylodon*, viz. *X.magnificus*, *X.pumilius* and *X.rickii*. Three new *Xylodon* species are described and illustrated, *X.ussuriensis* and *X.crystalliger* from East Asia and *X.attenuatus* from the Pacific Northwest America. The identity of *X.nongravis*, described from Sri Lanka, is discussed.

## Introduction

The genus *Lagarobasidium* was introduced by Jülich (1974) for three corticioid species, *L.cymosum* (D.P.Rogers & H.S.Jacks.) Jülich, *L.nikolajevae* (Parmasto) Jülich and *L.pruinosum* (Bres.) Jülich (the generic type). These species possess prominent, thin- or slightly thick-walled cystidia, suburniform tetrasporic basidia and thick-walled basidiospores. Eriksson and Ryvarden (1976) concluded that *L.pruinosum* is a later synonym of *Peniophoradetritica* Bourdot (Bourdot 1910), which prompted Jülich (1979) to move *P.detritica* to *Lagarobasidium*. At present, *L.detriticum* is accepted in a wide sense, with *Hyphodontiamagnacystidiata* Lindsey & Gilb., *H.nikolajevae* Parmasto and *Odontiapruinosa* Bres. as synonyms (http://www.mycobank.org [accessed 07 May 2018]).

Controversies over the taxonomic position of *Peniophoradetritica* emerged during the last decades. In modern morphology-based systems, it was first attributed to *Hyphodontia* J. Erikss., mainly due to hyphal characters and the shape of basidia (Eriksson 1958, Langer 1994). A second solution was introduced by Eriksson and Ryvarden (1976) who stressed the shape of cystidia and the thick-walled cyanophilous basidiospores and placed the species in *Hypochnicium*. The third option and the one chosen by Jülich (1974), was to place *P.detritica* in a genus of its own (Jülich 1974, 1979, Hjortstam and Ryvarden 2009).

Larsson et al. (2006) used the nrLSU and 5.8S genes for a phylogenetic analysis of Hymenochaetales and recovered *Peniophoradetritica* nested in a fairly well-supported clade that also included several species usually classified in *Hyphodontia*. This result supported the original opinion on relationships introduced by Eriksson (1958) but also showed that *Hyphodontia* sensu Eriksson was polyphyletic. The clade with *Peniophoradetritica*. recovered by Larsson et al. (2006), was later identified as *Xylodon*, type species *X.quercinus*, a genus that now covers the majority of species earlier referred to *Hyphodontia* (Hjortstam and Ryvarden 2009). On the other hand, Dueñas et al. (2009) studied sequences from the ITS region and concluded that molecular information supported recognition of the separate genus *Lagarobasidium*. These same ITS sequences have been used by several subsequent researchers, who therefore maintained *Lagarobasidium* separate from *Hyphodontia* sensu lato (Yurchenko and Wu 2014, Riebesehl et al. 2015, Chen et al. 2016, Chen et al. 2017, Kan et al. 2017, Riebesehl and Langer 2017, Yurchenko et al. 2017, Chen et al. 2018).

In the present study, we revise the *Lagarobasidiumdetriticum* complex based on morphological and molecular methods. We propose to consider *Lagarobasidium* as a later synonym of *Xylodon* and to restore *Odontiapruinosa* as an independent species. In addition, we describe three new *Xylodon* species and make five new combinations.

## Materials and methods

### Morphological methods

Type material and specimens from herbaria H, S, O, GB, BPI, TAAM and BAFC were studied. Herbarium abbreviations are given according to Index Herbariorum (Thiers). Microscopic methods are described in Miettinen et al. (2006). All measurements were made in Cotton Blue (CB, Merck 1275) with phase contrast illumination (1250×). The following abbreviations are used in microscopic descriptions: L – mean spore length; W – mean spore width; Q – mean L/W ratio; n – number of spores (hyphae, basidia) measured per number of specimens. We excluded 5% of measurements from each end of the range representing variation of basidiospores and cystidia. Excluded extreme values are given in parentheses when they differ substantially from the lower or higher 95% percentile.

### DNA extraction and sequencing

For DNA extraction we used either the standard CTAB protocol (Griffith and Shaw 1998) or DNeasy Plant Mini kit (Qiagen, Hilden, Germany). Primers ITS1F (Gardes and Bruns 1993), ITS4 (White et al. 1990) and LR21 (Hopple and Vilgalys 1999) were used to amplify the internal transcribed spacers 1 and 2 and the 5.8S gene. LR0R, LR5 (Moncalvo et al. 2002) and LR7 (Hopple and Vilgalys 1999) were used to amplify 28S large ribosomal subunit. Polymerase chain reaction (PCR) products were purified with the Cleanup Standard kit (Evrogen Ltd, Moscow, Russia) or QIAquick PCR purification kit (Qiagen, Hilden, Germany). Sequencing reactions were performed either by the Evrogen company (Moscow, Russia) following the BigDye terminator protocol (ABI Prism) on an Applied Biosystems 3730 xl automatic sequencer (Applied Biosystems, CA, USA) with primers ITS1F and ITS4 or with an external service provided by Macrogen (South Korea) using primers ITS1, ITS4, CTB6 (http://plantbio.berkeley.edu/~bruns/), LR5 and LR3R (Hopple and Vilgalys 1999).

### Phylogenetic analyses

DNA sequences were edited in Geneious (Biomatters Ltd, Auckland, New Zealand) or in Sequencher 5.2.4 (Gene Codes Co., Ann Arbor, MI, USA) and deposited in GenBank (Table 1). We compiled two sequence datasets. The first one contains full ITS sequences from 83 specimens. The second dataset includes ITS and nLSU sequences from 24 specimens and is a subset of the taxa in the ITS-only dataset. In both datasets, *Hastodontiahastata* (Litsch.) Hjortstam & Ryvarden (Hymenochaetales) was included as outgroup (Larsson et al. 2006). We generated 13 ITS and 6 nLSU sequences for this study; other sequences used in the analyses were downloaded from GenBank (Benson et al. 2018) or UNITE (Kõljalg et al. 2013) (Table 1). Alignments were calculated through MAFFT 7.407 online server (https://mafft.cbrc.jp/alignment/server/) using the L-INS-I strategy (Katoh et al. 2017) and then manually adjusted. The alignments are deposited in TreeBASE (http://purl.org/phylo/treebase/phylows/study/TB2:S23057).

**Table 1.** Specimens and GenBank and UNITE accession numbers for DNA sequences used in this study.

Species	Specimen voucher	GenBank or UNITE accession numbers for ITS	GenBank or UNITE accession numbers for LSU	Reference
*Hastodontiahastata* (Litsch.) Hjortstam & Ryvarden	Larsson 14646	MH638232	MH638232	this study
*Lyomycesallantosporus* Riebesehl, Yurchenko & E. Langer	FR-0249548, Holotype	KY800397	KY795963	Yurchenko et al. (2017)
*Lyomycescrustosus* (Pers.) P. Karst.	Larsson 11731	DQ873614	DQ873614	Larsson et al. (2006)
*Lyomyceserastii* (Saaren. & Kotir.) Hjortstam & Ryvarden	MA-Fungi 34,336	JX857800		Yurchenko et al. (2017)
*Lyomycesgriseliniae* (G. Cunn.) Riebesehl & E. Langer	Larsson 12971	DQ873651		Larsson et al. (2006)
*Lyomycesmascarensis* Riebesehl, Yurchenko & E. Langer	KAS-GEL4833, Holotype	KY800399	KY795964	Yurchenko et al. (2017)
*Lyomycesmicrofasciculatus* (Yurchenko & Sheng H. Wu) Riebesehl & E. Langer	TNM F24757, Holotype	JN129976		Yurchenko and Wu (2014)
*Lyomycesorganensis* Yurchenko & Riebesehl	MSK7247, Holotype	KY800403	KY795967	Yurchenko et al. (2017)
*Lyomycesorientalis* Riebesehl, Yurchenko & E. Langer	KAS-GEL3400	DQ340326	DQ340353	Yurchenko et al. (2017)
*Lyomycespruni* (Lasch) Riebesehl & E. Langer	Ryberg 021018	DQ873624	DQ873625	Larsson et al. (2006)
*Lyomycessambuci* (Pers.) P. Karst.	KAS-GEL2414	KY800398		Yurchenko et al. (2017)
	KAS-JR7	KY800402	KY795966	Yurchenko et al. (2017)
*Lyomycesvietnamensis* (Yurchenko & Sheng H. Wu) Riebesehl & E. Langer	TNM F973, Holotype	JX175044		Yurchenko and Wu (2014)
*Paliferverecundus* (G. Cunn.) Stalpers & P.K. Buchanan	Larsson 12261	DQ873642		Larsson et al. (2006)
*Xylodonapacheriensis* (Gilb. & Canf.) Hjortstam & Ryvarden	Canfield 180, Holotype	KY081800		Riebesehl and Langer (2017)
*Xylodonasperus* (Fr.) Hjortstam & Ryvarden	H6013167	UDB031926		Unpublished
	KG Nilsson s. n.	DQ873606	DQ873607	Larsson et al. (2006)
	UC2023169	KP814365		Riebesehl and Langer (2017)
*Xylodonastrocystidiatus* (Yurchenko & Sheng H. Wu) Riebesehl, Yurchenko & E. Langer	Wu 9211-71	JN129972	JN129973	Yurchenko and Wu (2014)
*Xylodonattenuatus* Spirin & Viner	Spirin 8775, Holotype	MH324476		this study
*Xylodonborealis* (Kotir. & Saaren.) Hjortstam & Ryvarden	Spirin 9416	MH317760	MH638259	this study
	TU115575	UDB016473		Unpublished
	UC2022850	KP814307		Riebesehl and Langer (2017)
	KUN2352	MH307753	MH638263	this study
	TU115495	UDB016350		Unpublished
	TU124171	UDB028164		Unpublished
*Xylodonbubalinus* (Min Wang, Yuan Y. Chen & B.K. Cui) C.C. Chen & Sheng H. Wu	Cui 12887	KY290982		Wang and Chen (2017)
*Xylodonchinensis* (C.C. Chen & Sheng H. Wu) C.C. Chen & Sheng H. Wu	Wu 1307-42	KX857802		Chen et al. (2017)
	Wu 1407-105, Holotype	KX857804		Chen et al. (2017)
*Xylodoncrystalliger* Viner	KUN2312, Holotype	MH324477		this study
*Xylodondetriticus* (Bourdot) Viner & Spirin	Zíbarová 30.10.17	MH320793	MH651372	this study
	Zíbarová 26.05.17	MH320794	MH638264	this study
*Xylodonflaviporus* (Berk. & M.A. Curtis ex Cooke) Riebesehl & E. Langer	ICMP13836	AF145585		Paulus et al. (2000)
*Xylodonhastifer* (Hjortstam & Ryvarden) Hjortstam & Ryvarden	Ryvarden 19767, Holotype	KY081801		Riebesehl and Langer (2017)
*Xylodonheterocystidiatus* (H.X. Xiong, Y.C. Dai & Sheng H. Wu) Riebesehl, Yurchenko & E. Langer	Wu 9209-27	JX175045		Yurchenko and Wu (2014)
*Xylodonlenis* Hjortstam & Ryvarden	Wu 0808-32	JX175043	KX857820	Yurchenko and Wu (2014)
	Wu 890714-3, Holotype	KY081802		Riebesehl and Langer (2017)
*Xylodonmollissimus* (L.W. Zhou) C.C. Chen & Sheng H. Wu	LWZ20160318-3, Holotype	KY007517		Kan et al. (2017)
*Xylodonnespori* (Bres.) Hjortstam & Ryvarden	B Nordén 030915	DQ873622		Larsson et al. (2006)
	GEL3158	DQ340310	DQ340346	Riebesehl and Langer (2017)
	GEL3290	DQ340309		Unpublished
	GEL3302	DQ340308		Unpublished
	GEL3309	DQ340307	DQ340345	Yurchenko and Wu (2014)
*Xylodonniemelaei* (Sheng H. Wu) Hjortstam & Ryvarden	GC 1508-146	KX857798		Chen et al. (2017)
	GEL4998	EU583422	DQ340348	Riebesehl and Langer (2017)
	Wu 1010-62	KX857799		Chen et al. (2017)
*Xylodonnongravis* (Lloyd) Spirin & Viner	CHWC1506-2	KX857800		Chen et al. (2017)
	Dai 11686	KT989968		Chen et al. (2017)
	GC1412-22	KX857801		Chen et al. (2017)
	Spirin 5763	MH324469	MH656724	this study
*Xylodonnothofagi* (G. Cunn.) Hjortstam & Ryvarden	PDD:91630	GQ411524		Fukami et al. (2010)
*Xylodonovisporus* (Corner) Riebesehl & E. Langer	ICMP13837	AF145587		Paulus et al. (2000)
	KUC20130725-29	KJ668513	KJ668365	Jang et al. (2016)
	Wu 0809-76	KX857803		Chen et al. (2017)
*Xylodonparadoxus* (Schrad.) Chevall.	FCUG 1517	AF145572		Paulus et al. (2000)
	FCUG 2425	AF145571		Paulus et al. (2000)
	Miettinen 7978	FN907912	FN907912	Miettinen and Larsson (2011)
*Xylodonpruinosus* (Bres.) Spirin & Viner	Larsson 14653	UDB024816		Unpublished
	Spirin 2877	MH332700		this study
	UC2023108	KP814412		Rosenthal et al. (2017)
*Xylodonpseudotropicus* (C.L. Zhao, B.K. Cui & Y.C. Dai) Riebesehl, Yurchenko & E. Langer	Dai 10768, Holotype	KF917543		Zhao et al. (2014)
*Xylodonquercinus* (Pers.) Gray	Kotiranta 27060	MH320792		this study
	Larsson 11076	KT361633	AY586678	Ariyawansa et al. (2015)
	Miettinen 15050,1	KT361632		Ariyawansa et al. (2015)
	Spirin 8565	MH316007		this study
	Spirin 8840	MH320791		this study
*Xylodonraduloides* (Pers.) Riebesehl & E. Langer	Dai 12631	KT203307	KT203328	Moncalvo et al. (2002)
	ICMP13833	AF145580		Paulus et al. (2000)
*Xylodonramicida* Spirin & Miettinen	Spirin 7664, Holotype	KT361634		Ariyawansa et al. (2015)
*Xylodonreticulatus* (C.C. Chen & Sheng H. Wu) C.C. Chen & Sheng H. Wu	GC 1512-1	KX857808		Chen et al. (2017)
	Wu 1109-178, Holotype	KX857805		Chen et al. (2017)
*Xylodonrhizomorphus* (C.L. Zhao, B.K. Cui & Y.C. Dai) Riebesehl, Yurchenko & E. Langer	Dai 12354	KF917544		Zhao et al. (2014)
*Xylodonrimosissimus* (Peck) Hjortstam & Ryvarden	CFMR:DLL2011-081	KJ140600		Brazee et al. (2014)
	Ryberg 021031	DQ873627	DQ873628	Larsson et al. (2006)
	UC2022842	KP814311		Rosenthal et al. (2017)
	UC2023109	KP814414		Rosenthal et al. (2017)
	UC2023147	KP814193		Rosenthal et al. (2017)
	UC2023148	KP814194		Rosenthal et al. (2017)
*Xylodonspathulatus* (Schrad.) Kuntze	GEL2690	KY081803		Riebesehl and Langer (2017)
	Larsson 7085	KY081804		Riebesehl and Langer (2017)
*Xylodonsubtropicus* (C.C. Chen & Sheng H. Wu) C.C. Chen & Sheng H. Wu	Wu 1508-2	KX857806		Chen et al. (2017)
	Wu 9806-105, Holotype	KX857807		Chen et al. (2017)
*Xylodonussuriensis* Viner	KUN1989, Holotype	MH324468		this study

We inferred phylogenetic trees with maximum likelihood (ML), maximum parsimony (MP) and Bayesian Inference (BI) but provide only the last one since all trees show congruity of the phylogenetic signal. Substitution models were determined with the aid of TOPALi 2.5 (Milne et al. 2008) based on Bayesian information criterion (BIC). GTR + G (nst = 6, rates = gamma) were the best-fit models for the whole ITS region in the ITS dataset as well as in the ITS + nrLSU dataset. SYM + G (nst = 6, rates = gamma, statefreqpr = fixed(equal)) was the best-fit model for the nrLSU region in the ITS + nrLSU dataset. The suggested models were implemented in the Bayesian phylogenetic analyses. We performed Bayesian inference with MrBayes 3.2 (Ronquist et al. 2012). In the analyses, three parallel runs with four chains each, temp = 0.2, were run for 3 million generations. All chains converged to <0.01 average standard deviation of split frequencies. A burn-in of 25% was used in the final analyses.

Maximum-likelihood (ML) analysis was performed in RAxML 7.2.8 (Stamatakis 2006) implemented in Geneious. Following models suggested by TOPALi 2.5, we preferred to use the GTR model with gamma correction (GTRGAMMA) in ML analysis for both datasets. The bootstrapping was performed using the ‘Rapid bootstrapping’ algorithm with the number of bootstrap replicates set as 1000.

Maximum parsimony (MP) analysis was performed using MEGA 7 (Kumar et al. 2016). We used the Subtree-Pruning-Regrafting (SPR) algorithm using all sites. The number of bootstrap replicates was set as 1000.

### Specimens examined (sequenced specimens are marked by an asterisk)

*Xylodonattenuatus*. USA. Washington: Clallam Co., La Push, *Pseudotsugamenziesii*, 8 Oct 2014, Spirin 8286a (H), Sol Duc, *Tsugaheterophylla*, 6 Oct 2014, Spirin 8133 (H); Jefferson Co., Hoh River, *Acermacrophyllum*, 20 Oct 2014, Spirin 8775* (H, holotype), *Tsugaheterophylla*, 20 Oct 2014, Spirin 8779 (H); Pend Oreille Co., Gypsy Meadows, *Piceaengelmannii*, 17 Oct 2014, Spirin 8694* (H). Canada. British Columbia: Fraser-Fort George Reg. Dist., Mt. Robson Provincial Park, *Picea sp*., 25 Jul 2015, Spirin 8900a (H).

*X.borealis*. Russia. Nizhny Novgorod Reg.: Lukoyanov Dist., Panzelka, *Quercusrobur* (very rotten log), 17 Aug 2015, Spirin 9416* (H).

*X.brevisetus*. Russia. Moscow: Losiny Ostrov Nat. Park, log of *Pinussylvestris*, 1 Oct 2016, A.Nechaev KUN2352* (H).

*X.crystalliger*. Russia. Primorie: Khasan Dist., Kedrovaya Pad Nat. Res., on angiosperm wood, 25 Jul 2016, I.Viner KUN 2312* (H, holotype); ibidem 29 Jul 2017, F.Bortnicov, KUN 3347 (H).

*X.detriticus*. Czech Republic. Karlovarský kraj: Sokolov, Antonín mine spoil, on *Phragmitesaustralis*, 26 May 2017, L.Zíbarová (H*); Liberecký kraj: Liberec, Uhelná, on *Calamagrostisepigejos*, 30 Oct 2017, L.Zíbarová (H*). France. Auvergne: Allier, St. Priest, on fern, 1 Sep 1909, H.Bourdot 7226 (S F204453, lectotype of *Peniophoradetritica*). Italy. Lazio: Circeo Nat. Park, on *Pinuspinea* bark, 23 Oct 1984, K.H.Larsson 5496 (GB); ibidem, on fallen leaves, 24 Oct 1984, K.H.Larsson 5622 (GB); ibidem, on ferns, 24 Oct 1984, K.H.Larsson 5627 (GB).

*X.magnificus*. Argentina. Tierra del Fuego: Ushuaia, Estancia Moat, on *Drimyswinteri*, 21 Mar 1998, A.Greslebin 1387 (GB, paratype duplicate).

*X.nongravis*. Russia. Khabarovsk Reg.: Khabarovsk Dist., Ulun, on *Salixschwerinii*, 25 Aug 2012, V.Spirin 5615 (H); ibidem, on *Corylusmandshurica*, 28 Aug 2012, V.Spirin 5763* (H); Primorie Reg.: Krasnoarmeiskii Dist., Melnichnoe, on *Corylusmandshurica*, 21–23 Aug 2013, V.Spirin 6218, 6260, 6281 (H). Sri Lanka. Peradeniya, on rotten branch, T.Petch (BPI US0305211, holotype of *Polyporusnongravis*).

*X.pruinosus*. Estonia. Ida-Virumaa: Kohtla-Järve, Pärnassaare, on *Betulapubescens*, 1 Oct 1958, E.Parmasto (TAAM, holotype of *Hyphodontianikolajevae*). Finland. Helsinki: Veräjämäki, on *Salixcaprea*, 4 Sep 2011, O.Miettinen 14651.4 (H). Germany. Nordrhein-Westfalen, on *Betula* sp., W.Brinkmann (S F204462, isolectotype of *Odontiapruinosa*). Norway. Akershus: Frogn, decaying deciduous wood, 3 Oct 2010, K.H.Larsson 14653* (O). Russia. Nizhny Novgorod Reg.: Bogorodsk Dist., Krastelikha, on *Quercusrobur*, 11 Aug 2009, V.Spirin 2877* (H); Lukoyanov Dist., Panzelka, on *Populustremula*, 19 Aug 2015, V.Spirin 9581 (H); Razino, on *Quercusrobur*, 16 Aug 2015, V.Spirin 9350 (H); Srednii, on *Tiliacordata*, 18 Aug 2006, V.Spirin 2601 (H); Pavlovo Dist., Chudinovo, on *Populustremula*, 3 Oct 2015, V.Spirin 9994 (H); Sverdlovsk Reg.: Nizhnisereginskii Dist., Olenii Ruchii Nat. Park, on *Populustremula*, 19–20 Aug 2002, H.Kotiranta 19684b, 19687, 19715a (H). USA. New York: Franklin County, Paul Smith’s, on *Populustremuloides*, 12 Sep 1965, R.L.Gilbertson 5481 (GB, isotype of *Hyphodontiamagnacystidiata*).

*X.pumilius*. Argentina. Chubut: Río Senguer, Lago La Plata, on *Nothofaguspumilio*, 26–28 Mar 1996, A.Greslebin 701 (GB, paratype duplicate).

*X.quercinus*. Canada. Alberta: Yellowhead Co., William A. Switzer Prov. Park, on *Populustremuloides*, 24 Jul 2015, V.Spirin 8840* (H). Finland. Uusimaa: Helsinki, Veräjänmäki, on angiosperm wood, 12 Apr 2008, O.Miettinen 12409* (H). Russia. Chukotka: Anadyr, on *Alnusfruticosa*, 19 Sep 2009, H.Kotiranta 27060* (H). USA. Washington: Pend Oreille Co., Slate Creek, on *Coryluscornuta*, 15 Oct 2014. V.Spirin 8565* (H).

*X.rickii*. Brazil. Rio Grande do Sul: S. Salvador, 5 Apr 1944, J.Rick 20847 (O, isotype of *Odontiapolycystidifera*).

*X.ussuriensis*. Russia. Primorie: Khasan Dist., Kedrovaya Pad Nat. Res., angiosperm wood, 24 Jul 2016, I.Viner KUN 1989* (H, holotype of *Xylodonussuriensis*), I.Viner KUN 2103, 2186.

## Results

**Figure 1.**
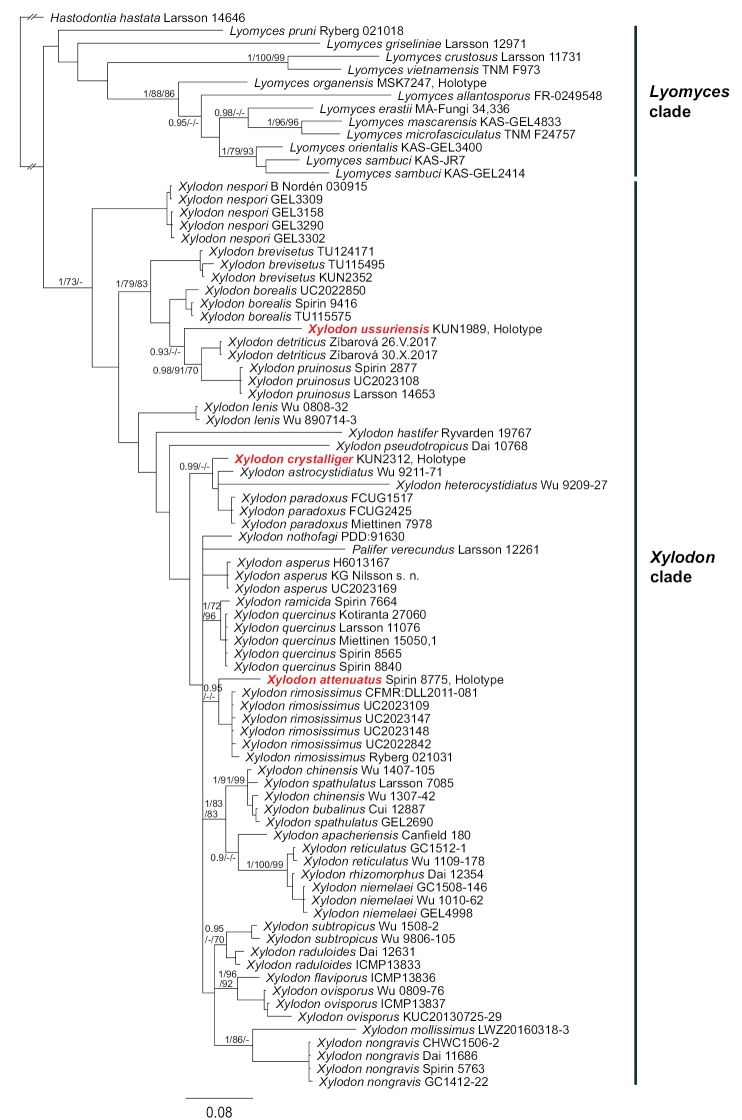
Phylogenetic relationships of *Xylodon* inferred from ITS sequences using Bayesian analysis. A 50% majority rule consensus phylogram. Bayesian posterior probabilities, ML bootstrap and MP bootstrap values are shown on nodes; branch lengths reflect estimated number of changes per site.

**Figure 2.**
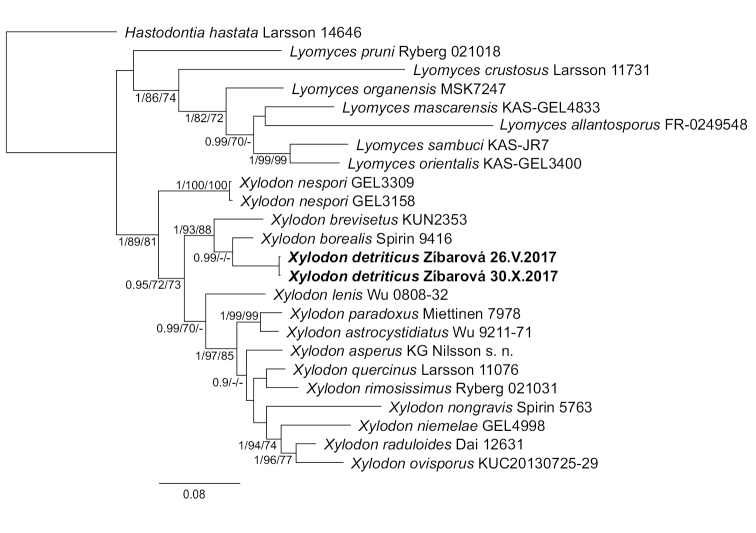
Phylogenetic relationships of *Xylodon* inferred from ITS and LSU sequences using Bayesian analysis. A 50% majority rule consensus phylogram. Bayesian posterior probabilities, ML bootstrap and MP bootstrap values are shown on nodes; branch lengths reflect estimated number of changes per site.

For both datasets, the Bayesian inference returned trees with two main clades (Figures 1, 2); the largest clade is well-supported and corresponds to *Xylodon* (pp 1.0), while the other clade is unsupported and includes *Lyomyces*, the *Hyphodontiacrustosa* group, *H.pruni* and *Rogersellagriseliniae* (pp 0.89). Basal relationships within *Xylodon* are not resolved. *Peniophoradetritica* and its allied species are nested within *Xylodon* and form a well-supported subclade together with *X.borealis* and *X.brevisetus* (Figures 1, 2). Maximum likelihood and maximum parsimony returned similar topologies and relevant support values from these analyses are indicated on nodes in Figures 1, 2.

In the ITS-only tree, three terminal branches represent new species that are described below. *Xylodonattenuatus* occurs as a sister taxon to *X.rimosissimus*; *X.crystalliger* forms a subclade with *X.astrocystidiatus*, *X.paradoxus* and *X.heterocystidiatus*; and *X.ussuriensis* is the sister taxon to *X.detriticus* and *X.pruinosus* (Figure 1).

The results allow us to introduce new species and new combinations as follows.

### Xylodon attenuatus

Taxon classificationFungiHymenochaetalesSchizoporaceae

Spirin & Viner sp. nov.

MB825367

Figure 3

#### Type.

USA. Washington: Jefferson Co., Hoh River, on *Acermacrophyllum*, 20 Oct 2014, V.Spirin 8775 (H) – ITS sequence, GenBank MH324476.

#### Etymology.

Attenuatus (lat., adj.) – exhausted, thin.

#### Description.

Basidiocarp effused, up to 5 cm in widest dimension. Sterile margin white, up to 1 mm wide. Hymenial surface cream-coloured, grandinioid to odontoid; projections rather regularly arranged, from 80 µm to 200 μm high, 70–90 μm broad at base, 6–8(–9) per mm. Hyphal structure monomitic, hyphae clamped, cyanophilous. Subicular hyphae densely interwoven, thin-walled, (2–)2.4–4.6 μm in diam. (n=60/6), often short-celled, the outline of these hyphae often irregular. Tramal hyphae subparallel, thin-walled, in subhymenium densely arranged, sometimes short-celled, 2.4–3.6 μm in diam. (n=62/6). Large stellate crystals 10–13.3 μm in diam. present in subiculum and trama. Cystidia originating from subhymenium, of two types: a) subcapitate or capitate cystidia, (12–)13.5–25.1(–37)×(2.7–)3.3–5(–5.5) μm (n=80/6), b) hyphoid cystidia, (14–)16–38.3(–40.8)×2.8–4.5 (n=51/6), sometimes with crystalline cap on the top; some cystidia with granular contents in CB. Basidia suburniform, 4-spored, (12.2–)14–22(–25)×(3–)3.3–4.6(–5) μm (n=61/2), slightly thick-walled at the base. Basidiospores thin-walled, ellipsoid, (3.7–)4.1–5.5(–6)×(3–)3.4–4.5(–4.9) μm (n=180/6), L=4.85, W=3.98, Q=1.22, slightly cyanophilous.

**Figure 3.**
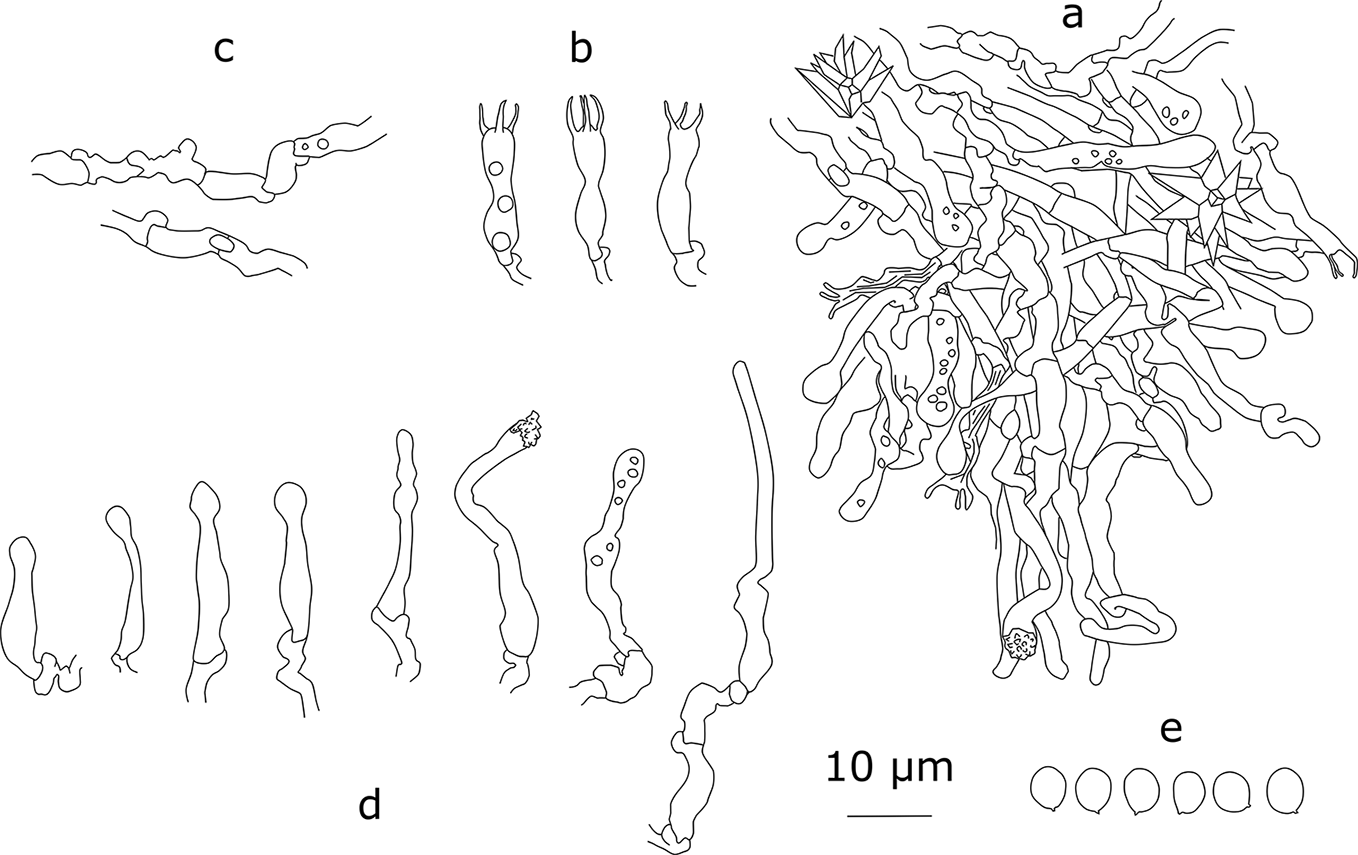
*Xylodonattenuatus* (holotype): **a** section through an aculeus **b** basidia **c** subhymenial short-celled hyphae **d** cystidia **e** basidiospores.

#### Distribution and ecology.

North-western USA (Washington), on angiosperm and gymnosperm wood (fallen decorticated logs).

#### Remarks.

*Xylodonattenuatus* bears morphological similarity to *X.borealis*, although densely arranged hyphae, star-like crystals and a regular presence of cystidia with granular contents make it easily recognisable. The crystalline caps on hyphoid cystidia are other characteristics useful for the identification of *X.attenuatus*.

### Xylodon crystalliger

Taxon classificationFungiHymenochaetalesSchizoporaceae

Viner sp. nov.

MB825368

Figure 4

#### Type.

RUSSIA. Primorie: Khasan Dist., Kedrovaya Pad Nat. Res., on angiosperm wood, 25 Jul 2016, I.Viner KUN 2312 (H) – ITS sequence, GenBank MH324477.

#### Etymology.

Crystalliger (lat., adj.) – bearing crystals.

#### Description.

Basidiocarp effused, soft membranaceous, up to 6 cm in widest dimension. Sterile margin poorly defined, up to 0.3 mm wide. Hymenial surface white, minutely odontioid, i.e. covered by small peg-like hyphal projections up to 60–100 μm high, 60–75 μm broad at base, 10–15 per mm, with flattened fimbriate apices. Surface between projections porulose-reticulate. Hyphal structure monomitic, hyphae clamped, faintly cyanophilous. Subicular hyphae densely interwoven, often with thickened walls, 3.2–4.4 μm in diam. (n=20/2), smooth or sparsely encrusted. Tramal hyphae subparallel, thin- to clearly thick-walled, sparsely encrusted, subhymenial hyphae densely arranged, sometimes short-celled, 2.5–3.2 μm in diam. (n=20/2), sparsely encrusted. Hyphal ends at the top of projections often strongly encrusted. Cystidia of two types: a) sparsely encrusted hyphoid cystidia at the top of projections, 21.0–29.0×2.9–4.1(–4.4) μm (n=40/2), b) subcapitate or cylindrical cystidia, of subhymenial origin, rather variable in shape and size, (11.8–)14.1–25.0(–28.0)×(2.6–)2.9–4.6(–4.8) μm (n=40/2), often heavily encrusted and rarely with a stellate crystalline cap 3.5–4.5 μm in diam. Basidia suburniform, 4-spored, 13.4–18.4(–19.0)×4.2–4.7 μm (n=20/2), slightly thick-walled at the base. Basidiospores thin-walled, elliptical, occasionally with an oil-drop, (3.1–)4.2–5.1(–5.9)×(2.4–)3.3–4.2 μm (n=60/2), L=4.66, W=3.71, Q=1.26, slightly cyanophilous.

**Figure 4.**
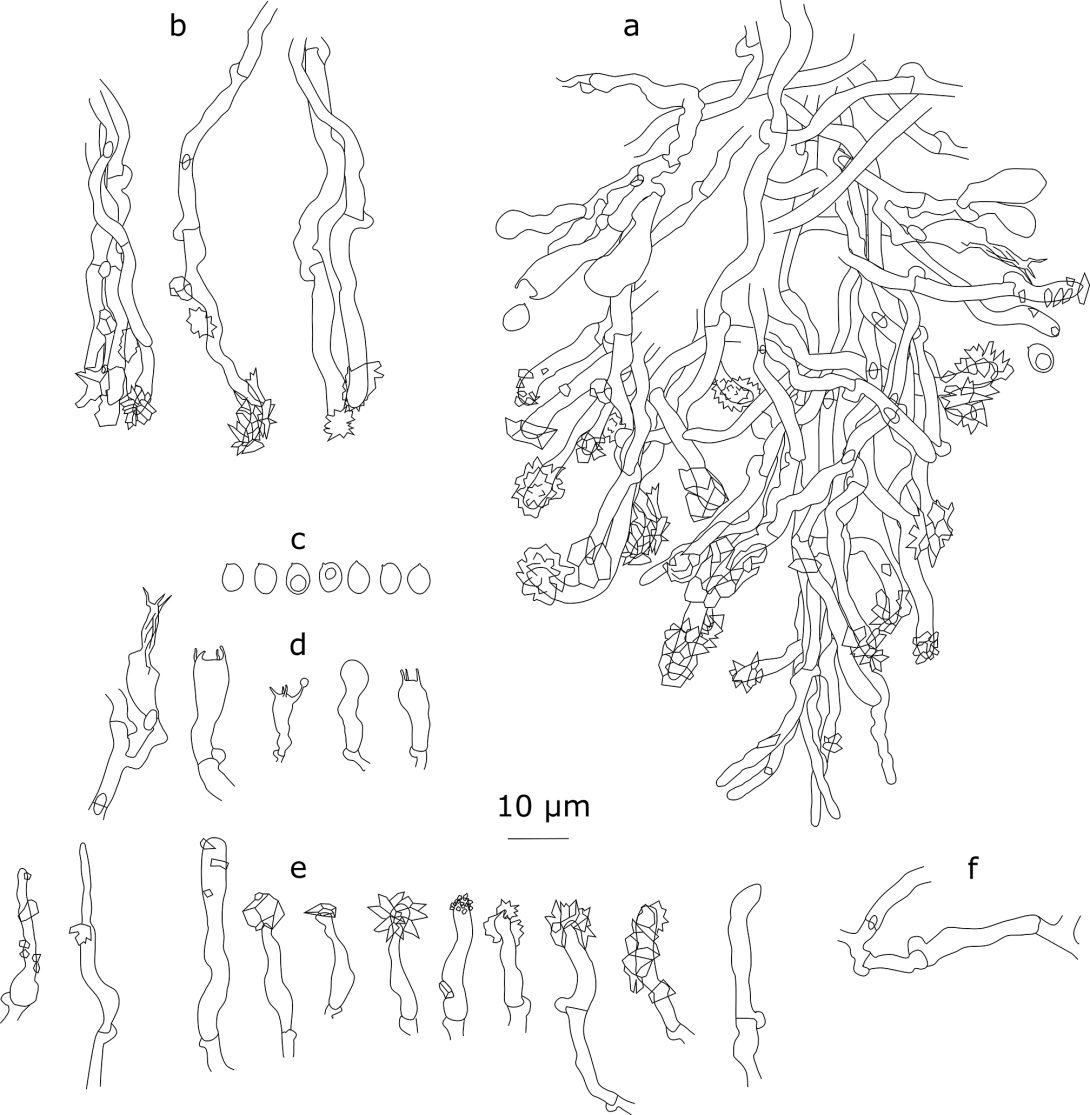
*Xylodoncrystalliger* (holotype): **a** section through an aculeus **b** apically encrusted hyphae from aculeal tips **c** basidiospores **d** basidia **e** cystidia **f** subhymenial hyphae.

#### Distribution and ecology.

East Asia (Russian Far East), on decayed angiosperm logs.

#### Remarks.

The peg-like hymenial projections and cystidia with stellate caps are characteristic for *X.crystalliger* and make it reminiscent of *Xylodonastrocystidiatus* (Yurchenko & Sheng H. Wu) Riebesehl, Yurchenko & Langer. The latter species is known from Taiwan and differs from *X.crystalliger* by having longer basidiospores and presence of constricted and bladder-like hymenial cystidia.

### Xylodon detriticus

Taxon classificationFungiHymenochaetalesSchizoporaceae

(Bourdot) K.H. Larss., Viner & Spirin comb. nov.

MB825366

Figures 5 , 6c , 7

#### Basionym.

*Peniophoradetritica* Bourdot, Revue Scientifique du Bourbonnais et du Centre de la France 23: 13. 1910. ≡ *Lagarobasidiumdetriticum* (Bourdot) Jülich, Persoonia 10: 334. 1979. Type. France. Auvergne: Allier, St. Priest, fern, 1.IX.1909 Bourdot 7226 (lectotype S! [F204453], designated by Eriksson and Ryvarden 1976: 703).

#### Description.

Basidiocarps effused, up to 5 cm in widest dimension. No differentiated margin. Hymenial surface white, smooth or warted, farinaceous. Hyphal structure monomitic, hyphae clamped, faintly cyanophilous, thin-walled. Subicular hyphae interwoven and frequently branched, (2.2–)3.0–5.9 μm in diam. (n=61/6). Tramal hyphae subparallel, subhymenial hyphae short-celled, (1.5–)1.9–3.5 μm in diam. (n=61/6). Large, rhomboid or stellate crystals abundant in trama and subiculum, 8–10.5 μm in diam. Cystidia of two types: a) large, thin-walled cystidia of subicular or tramal origin, cylindrical or clavate, rarely slightly thick-walled (wall not exceeding 1 μm thick), (30.0–)58.9–110.0(–115.0)×4.1–8.5(–9.6) μm (n=120/6), occasionally bearing 1–2 clamped septa, b) rare astrocystidia of subhymenial origin, with a stellate crystalline cap 10–23×2–3.1 μm, in some specimens difficult to find. Basidia suburniform, 4-spored, (12.2–)13.1–20.0×(3.1–)3.4–5.0 μm (n=61/6), thin-walled. Basidiospores clearly thick-walled, elliptical to broadly elliptical, usually with an oil-drop, (3.3–)4.3–5.7(–6.1)×3.2–4.1(–4.5) μm (n=190/6), L=4.92, W=3.69, Q=1.34, cyanophilous.

**Figure 5.**
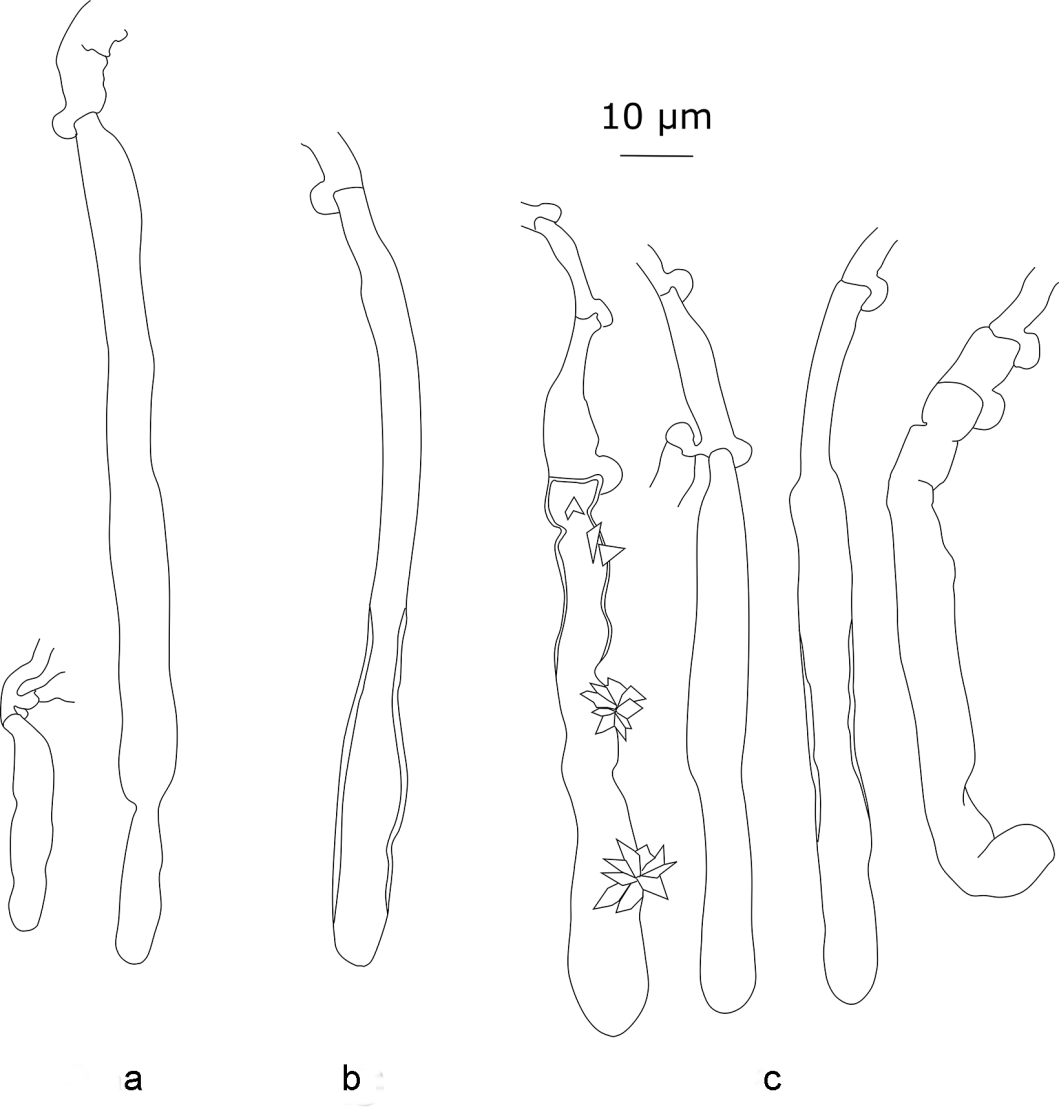
Cystidial elements of *Xylodondetriticus*: **a** Larsson 5496 **b** Zíbarová 26.V.2017 **c** Zíbarová 30.X.2017.

#### Distribution and ecology

. Europe (Czech Republic, France, Italy), on herbaceous remnants, once collected from pine bark at the same spot where it was found on fern remains.

#### Remarks.

**Figure 6.**
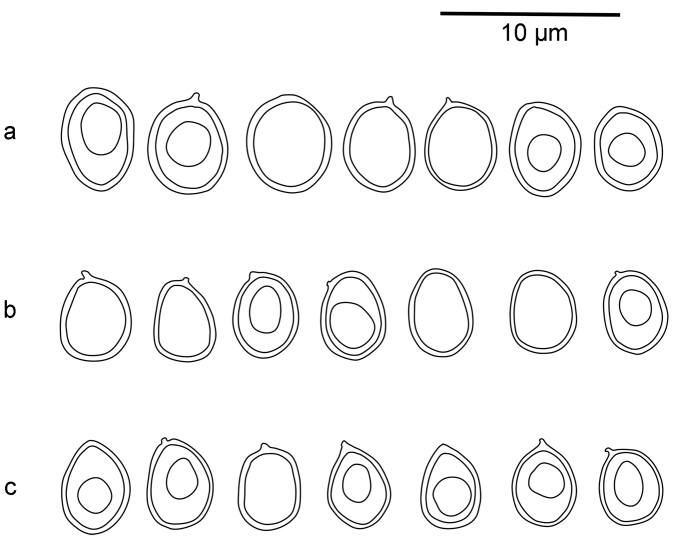
Basidiospores of two *Xylodon* species in CB: **a***X.pruinosus* (Spirin 9994) **b***X.pruinosus* (isotype of *Hyphodontiamagnacystidiata*) **c***X.detriticus* Zíbarová (26.V.2017).

Eriksson and Ryvarden (1976) selected Bourdot 7226 (in herb. S) as lectotype. They also treated *Hyphodontianikolajevae* and *Odontiapruinosa* as synonyms. However, the type specimens of *H.nikolajevae* and *O.pruinosa* reveal small differences from the type material and other collections of *X.detriticus* studied by us. The main features of *X.detriticus* versus the two other taxa are narrower basidiospores (must be observed in cotton blue) and longer, narrower cystidia having no distinct intercalary inflation (Tables 2, 3, Figures 5, 6). Eriksson and Ryvarden (1976) attributed the differences in cystidia morphology between Bourdot’s specimen and types of *H.nikolajevae* and *O.pruinosa* to different stages of basidiocarp development. Our investigation indicates that the differences are genetic and species specific. Differences in basidiospore size and shape are detectable in CB but not in KOH, which could explain why they have gone unnoticed in earlier studies.

Hjortstam and Ryvarden (2009) added *Hyphodontiamagnacystidiata* to the synonymy of *X.detriticus*. This species is, as far as we know, only known from the type, collected on dead wood of *Populus* in New York, USA (Lindsey and Gilbertson 1977). It has an odontioid basidiocarp and its cystidia are similar to those of *X.pruinosus* (Table 3, Figures 6, 8). On the other hand, the basidiospore size is very close to *X.detriticus* (Table 2). In the absence of sequenced material, it is not possible to decide whether this is an independent species or not. Considering that the single specimen was growing on wood and that *X.detriticus* is not yet found in North America, we prefer to keep *H.magnacystidiata* as a synonym of *X.pruinosus* (see below).

**Figure 7.**
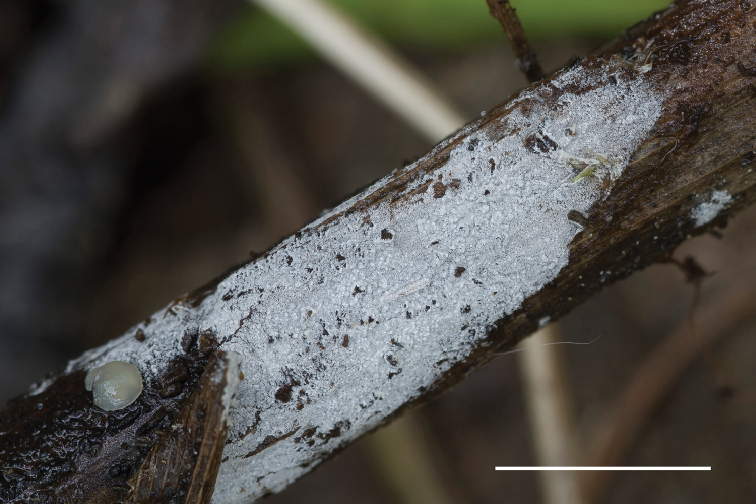
Basidiocarp of *Xylodondetriticus* (Zíbarová 26.V.2017). Scale bar: 5 mm.

**Figure 8.**
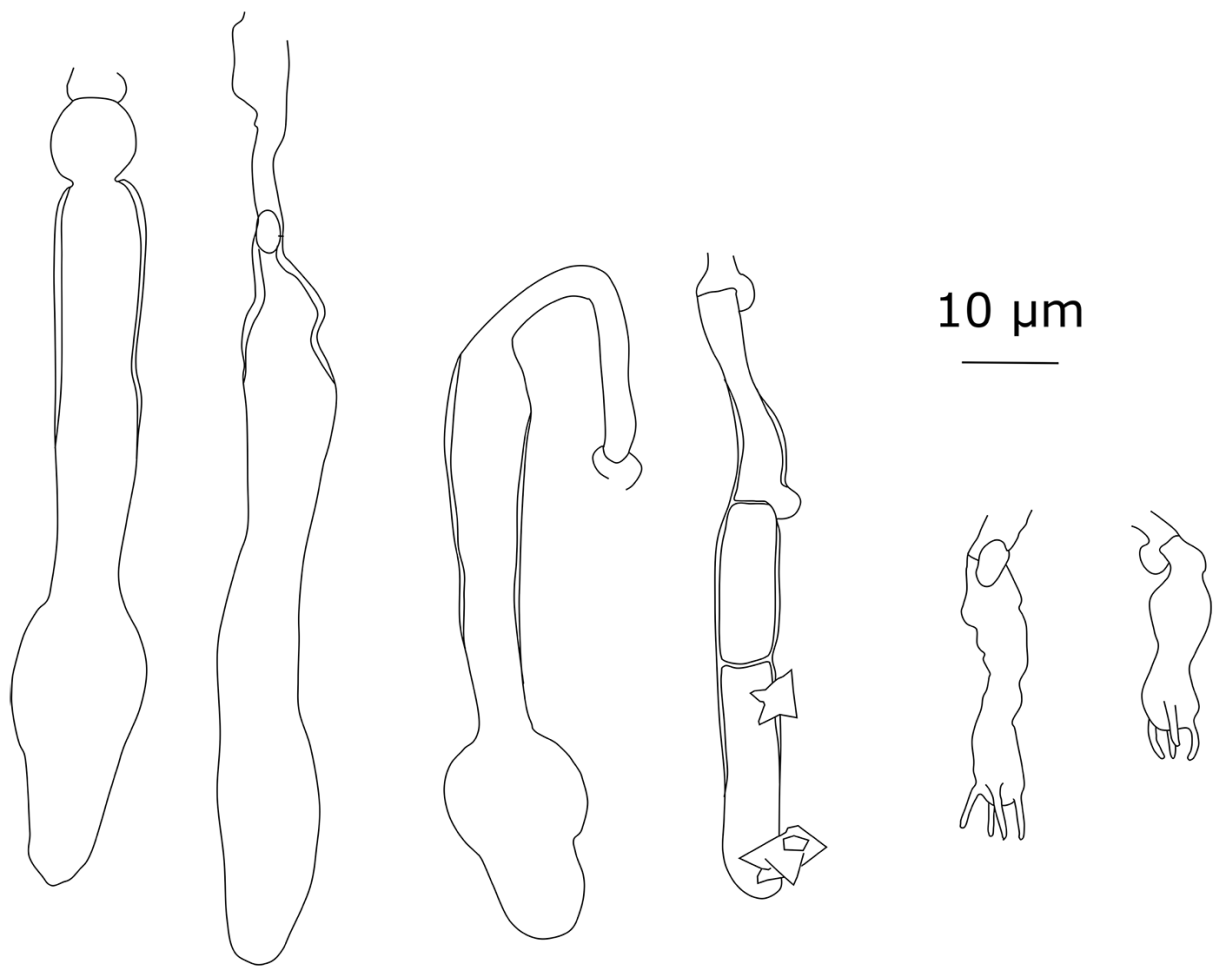
Cystidial elements and basidia of *Xylodonpruinosus* (isotype of *Hyphodontiamagnacystidiata*).

**Table 2.** Spore measurements of five *Xylodon* species.

Species / specimen	L'	L	W'	W	Q'	Q	n
* Xylodon attenuatus *	(3.7) 4.1–5.5 (6)	4.85	(3) 3.4–4.5 (4.9)	3.98	(0.98) 1.06–1.38 (1.46)	1.22	180
Holotype	(4.3) 4.4–5.7 (5.8)	4.86	(3) 3.5–4.3 (4.7)	3.84	(1.1) 1.2–1.4 (1.5)	1.27	30
Spirin 8133	(4.4) 4.54–5.3 (5.5)	5.01	(3.2) 3.8–4.6 (4.7)	4.14	(1.06) 1.1–1.33 (1.38)	1.21	30
Spirin 8286	(4.1) 4.14–5.74 (6)	4.98	(3.1) 3.84–4.5 (4.5)	4.11	(1.02) 1.09–1.34 (1.36)	1.21	30
Spirin 8779	(4) 4–5.2 (5.4)	4.67	(3) 3.2–4.3 (4.4)	3.82	(0.98) 1.04–1.38 (1.43)	1.23	30
Spirin 8900a	(3.7) 3.95–5.25 (5.6)	4.56	(3.4) 3.4–4.35 (4.9)	3.94	(1.02) 1.02–1.29 (1.37)	1.16	30
Spirin 8964	(4.5) 4.6–5.6 (5.7)	5.02	(3.5) 3.6–4.3 (4.8)	4.04	(1.1) 1.1–1.4 (1.4)	1.25	30
* Xylodon crystalliger *	(3.1) 4.2–5.1 (5.9)	4.66	(2.4) 3.3–4.2 (4.3)	3.71	(1) 1.1–1.4 (1.6)	1.26	60
Holotype	(3.1) 4.2–5.1 (5.9)	4.63	(2.4) 3.1–3.8 (3.9)	3.5	(1.2) 1.2–1.5 (1.6)	1.32	30
Bortnicov KUN 3347	(4.2) 4.2–5.3 (5.5)	4.69	(3.3) 3.6–4.2 (4.3)	3.91	(1) 1.1–1.4 (1.4)	1.2	30
* Xylodon detriticus *	(3.3) 4.3–5.7 (6.1)	4.92	(3.1) 3.2–4.1 (4.5)	3.69	(0.7) 1.1–1.6 (1.8)	1.34	190
Lectotype	(4.2) 4.3–6 (6.1)	5.07	(3.1) 3.2–4 (4.1)	3.59	(1.2) 1.2–1.6 (1.7)	1.42	39
Larsson 5496	(3.3) 4.2–5.5 (6)	4.87	(3.1) 3.2–4.1 (4.5)	3.61	(0.7) 1.1–1.6 (1.8)	1.36	30
Larsson 5622	(4) 4.2–5.1 (5.5)	4.6	(3.3) 3.4–3.9 (4)	3.63	(1.1) 1.1–1.4 (1.5)	1.27	30
Larsson 5627	(4) 4.2–5 (5.6)	4.69	(3.3) 3.3–4.1 (4.2)	3.73	(1.1) 1.2–1.4 (1.4)	1.26	31
Zibarova 26.V.2017	(4.4) 4.7–5.8 (5.9)	5.26	(3.2) 3.3–4.2 (4.3)	3.83	(1.1) 1.2–1.6 (1.7)	1.38	30
Zibarova 30.X.2017	(4.2) 4.2–5.7 (5.9)	4.99	(3.2) 3.3–4.1 (4.2)	3.78	(1.1) 1.1–1.5 (1.7)	1.32	30
* Xylodon pruinosus *	(4) 4.5–5.9 (7)	5.09	(3.3) 3.7–4.8 (5.7)	4.12	(0.8) 1.1–1.4 (1.5)	1.24	192
Holotype of *Hyphodontianikolajevae*	(4.6) 4.7–6 (7)	5.26	(3.5) 3.8–5 (5.3)	4.32	(1) 1.1–1.4 (1.4)	1.22	31
Holotype of *Odontiapruinosa*	(4) 4.1–5.7 (5.9)	4.95	(3.5) 3.6–4.5 (4.6)	4.03	(1.1) 1.1–1.4 (1.4)	1.23	40
Spirin 2877	(4.5) 4.7–6.1 (6.3)	5.28	(3.5) 3.8–5 (5.2)	4.21	(1) 1.1–1.4 (1.5)	1.26	30
Spirin 9350	(4.4) 4.7–5.7 (6.2)	5.21	(3.5) 3.8–4.8 (5.7)	4.17	(0.8) 1.1–1.4 (1.5)	1.26	31
Spirin 9581	(4.2) 4.2–5.8 (6.1)	4.99	(3.3) 3.6–4.4 (4.6)	3.98	(1) 1.1–1.4 (1.4)	1.25	30
Spirin 9994	(4.2) 4.6–5.1 (5.3)	4.89	(3.5) 3.6–4.5 (4.6)	4.04	(1.1) 1.1–1.3 (1.4)	1.21	30
Holotype of *Hyphodontiamagnacystidiata*	(4) 4.3–5.5 (5.6)	4.92	(3.1) 3.1–4 (4.2)	3.68	(1.1) 1.1–1.6 (1.7)	1.35	30
* Xylodon ussuriensis *	(4.8) 5.1–6 (6.2)	5.48	(3.7) 3.8–4.6 (4.8)	4.21	(1.2) 1.2–1.4 (1.5)	1.3	92
Holotype	(4.9) 5.1–5.9 (6.2)	5.48	(3.7) 3.8–4.6 (4.8)	4.22	(1.2) 1.2–1.4 (1.4)	1.3	32
Viner KUN 2103	(4.8) 5–6.1 (6.2)	5.6	(3.8) 3.8–4.7 (4.7)	4.24	(1.2) 1.2–1.4 (1.5)	1.32	30
Viner KUN 2186	(5) 5–5.7 (5.8)	5.37	(3.8) 4–4.5 (4.6)	4.18	(1.2) 1.2–1.4 (1.5)	1.28	30

**Table 3.** Measurements of cystidial elements of *Xylodondetriticum* and *X.pruimosus*.

Species / specimen	L'	L	W'	W	n
* Xylodon detriticus *	(30) 58.9–110 (115)	85	(4) 4.1–8.5 (9.6)	6.3	120
Lectotype	(67) 69.9–96.7 (110)	83.8	(4) 4–9.1 (9.2)	6.5	20
Larsson 5496	(30) 45.2–108.2 (112)	81.2	(4.1) 4.3–7 (7.2)	5.7	20
Larsson 5622	(30) 45–103 (110)	82.7	(4.1) 4.3–7.5 (8.5)	5.7	20
Larsson 5627	(56) 58.7–104.6 (110)	79.1	(4.4) 4.8–8.9 (9.6)	6.4	20
Zibarova 26.V.2017	(80) 83.8–103.3 (110)	95.1	(4) 5.4–8.1 (8.5)	7.1	20
Zibarova 30.X.2017	(67) 73.7–112.2 (115)	87.7	(4) 5–7.4 (7.5)	6.3	20
* Xylodon pruinosus *	(35) 44–84 (107)	61.9	(4) 4.9–10.9 (12.4)	7.2	146
Holotype of *Hyphodontianikolajevae*	(41) 43–95 (99)	64	(4) 5–12 (12)	7.7	21
Isolectotype of *Odontiapruinosa*	(43) 45.9–80.4 (107)	64	(4.6) 5.3–10.6 (12.4)	7.3	20
Spirin 2877	(35) 42.6–80 (80)	58.4	(4) 4.8–7.9 (8)	6.2	20
Spirin 9350	(41) 44.8–83.2 (86)	61.8	(4.6) 4.7–10 (10.7)	7.2	20
Spirin 9581	(49) 51.8–84.1 (86)	64.6	(4.9) 5–9 (11)	7.1	20
Spirin 9994	(45) 45.8–75.3 (81)	58.9	(5.3) 5.6–10.2 (10.8)	7.8	20
Isotype of *Hyphodontiamagnacystidiata*	(48) 51–95 (104)	75.8	(4.1) 6–12 (14)	8.4	25

*Xylodondetriticus* grows on ferns and grasses, developing thin farinaceous basidiocarps. The species evidently has a more southern distribution than *X.pruinosus*. Earlier reports of *X.detriticus* from woody substrates should be treated with caution and may represent *X.pruinosus* or as yet undescribed taxa.

### Xylodon magnificus

Taxon classificationFungiHymenochaetalesSchizoporaceae

(Gresl. & Rajchenb.) K.H. Larss. comb. nov.

MB827074

#### Basionym.

*Hyphodontiamagnifica* Gresl. & Rajchenb., Mycologia 92: 1160. 2000.

#### Type.

Argentina. Tierra del Fuego: Dpto. Ushuaia, Estancia Moat, on *Drimyswinteri*, 21 Mar 1998, M. Rajchenberg 11370 (holotype: BAFC [50038], by original designation).

For a detailed description and illustration, see Greslebin and Rajchenberg (2000). The authors compared the new species with *Xylodondetriticus* (as *Hyphodontiadetritica*) and *Hypochniciumrickii*. Our investigation of authentic material confirms the morphological similarity amongst these three species.

### Xylodon nongravis

Taxon classificationFungiHymenochaetalesSchizoporaceae

(Lloyd) C.C. Chen & Sheng H. Wu, in Chen et al. 2018: 349

Figure 9

#### Basionym.

*Polyporusnongravis* Lloyd, Mycol. Writings 6 (61): 891. 1919.

#### Type.

Sri Lanka. Peradeniya, on rotten branch, T.Petch (holotype BPI [305211]).

Wu (2000) re-described and illustrated this poroid species as *Hyphodontianongravis* (Lloyd) S.H. Wu. Our specimens collected in the Russian Far East fit well with his description. One of these collections (Spirin 5763) was sequenced and proved to be close to other sequences of *H.nongravis* available in GenBank. The species undoubtedly belongs to the core *Xylodon* clade (Figure 1) where it has been combined by Chen et al. (2018). However, the type specimen of *Polyporusnongravis* possesses small but clear morphological differences from our collections: in particular, wider pores (2–3 per mm in the type, 3–4 per mm in East Asian specimens) and broader tramal hyphae (4–6 μm vs. 3–4.5 μm in diam.), as well as broader, predominantly subglobose basidiospores, 3.9–4.7×3.6–4.2 μm (n=30/1), L=4.27, W=3.97, Q=1.08 (vs ovoid-ellipsoid, 4.0–5.2×3.0–4.1 μm (n=60/2), L=4.74, W=3.46, Q=1.38 in East Asian specimens). An epitype for *P.nongravis* from the *locus classicus* is needed to re-introduce this species based on modern methods and to clarify the taxonomic status of *X.nongravis* sensu East Asia.

**Figure 9.**
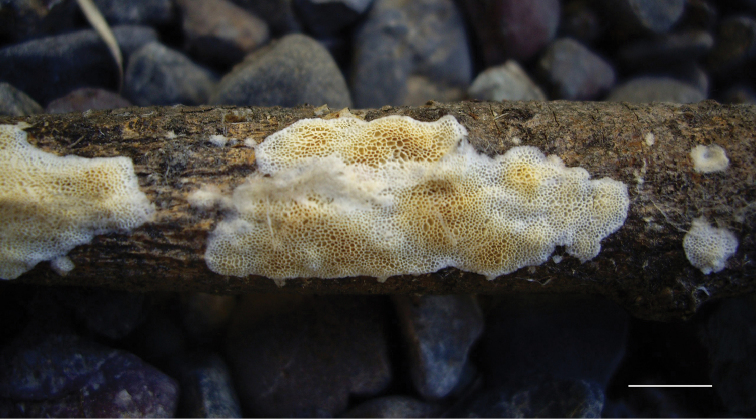
Basidiocarp of *Xylodonnongravis* (Spirin 5763). Scale bar: 5 mm.

### Xylodon pruinosus

Taxon classificationFungiHymenochaetalesSchizoporaceae

(Bres.) Spirin & Viner comb. nov.

MB825369

**Figure 10.**
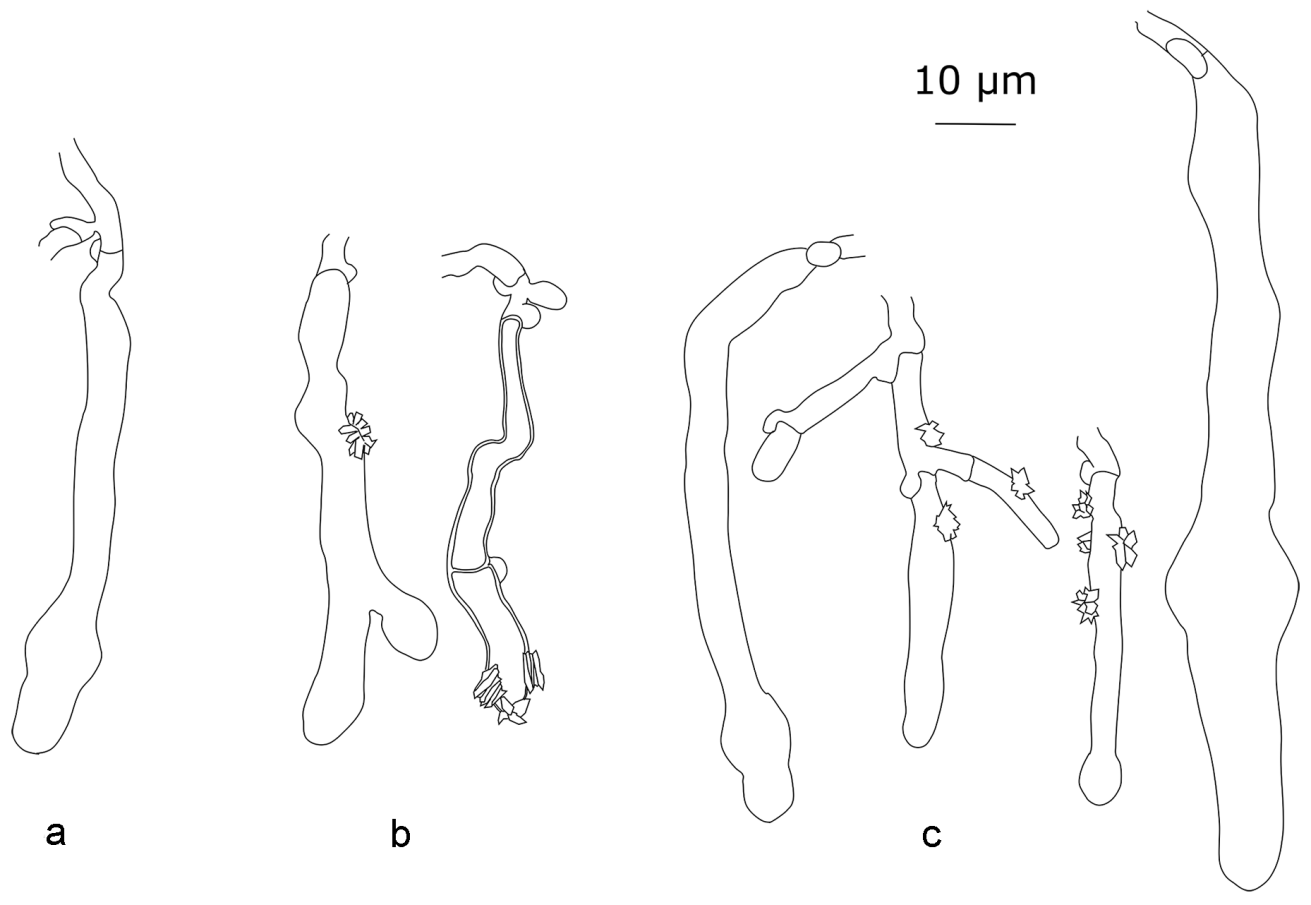
Cystidial elements of *Xylodonpruinosus*: **a** Spirin 9581 **b** Spirin 2877 **c** holotype of *Hyphodontianikolajevae*.

**Figure 11.**
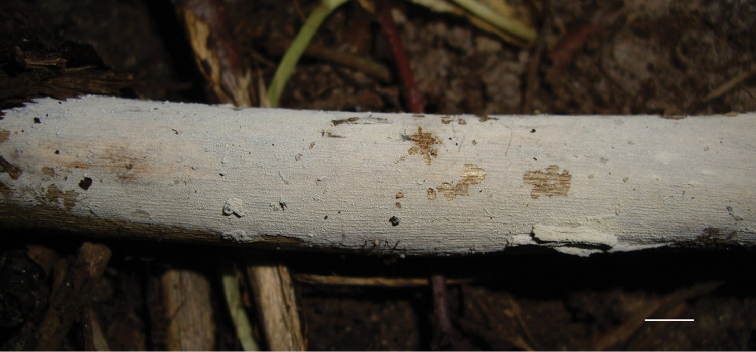
Basidiocarp of *Xylodonpruinosus* (Spirin 2877). Scale bar: 5 mm.

Figures 6 a,b , 8 , 10 , 11

#### Basionym.

*Odontiapruinosa* Bres., Annales Mycologici 18 (1–3): 43. 1920. ≡ *Lagarobasidiumpruinosum* (Bres.) Jülich, Persoonia 8: 84. 1974.

#### Type.

Germany. Nordrhein-Westfalen, Lengerich, W.Brinkmann (lectotype L [L 0053271], designated by Jülich 1974: 84).

= *Hyphodontianikolajevae* Parmasto, Conspectus Systematis Corticiacearum: 213. 1968. Type: Estonia. Ida-Virumaa, Kohtla-Järve, Pärnassaare, on *Betulapubescens*, 1 Oct 1958, E.Parmasto (holotype: TAAM [9683], by original designation).

= *Hyphodontiamagnacystidiata* Lindsey & Gilb., Mycotaxon 5: 315. 1977. Type: USA. New York, Franklin County, Paul Smith’s, on *Populustremuloides*, 12 Sep 1965, R.L.Gilbertson 5481 (holotype: BPI [266395], by original designation).

#### Description.

Basidiocarps annual, resupinate, up to 5 cm in widest dimension. Margin poorly differentiated, pruinose. Hymenial surface greyish-white or pale cream-coloured, grandinioid to odontoid; projections rather regularly arranged, from 100 µm to 250 µm high, 80–100 μm broad at base, 6–8 per mm. Hyphal structure monomitic, hyphae clamped, faintly cyanophilous, thin-walled. Subicular hyphae interwoven and frequently branched, 2.2–4.7(–6.1) μm in diam. (n=60/6). Tramal hyphae subparallel, subhymenial hyphae short-celled, 2.0–3.5(–3.9) μm in diam. (n=60/6). Stellate crystals abundant in trama, subiculum and subhymenium, 4.4–8.3 μm in diam. Cystidia large, thin-walled, of subicular, tramal or subhymenial origin, clavate to spathuliform, often with an intercalary inflation, sometimes slightly thick-walled (wall not exceeding 1 μm thick), rarely forked, (35.0–)44.0–84.0(–107.0)×(4.0–)4.9–10.9(–12.4) μm (n=121/6), occasionally bearing 1–2 clamped septa. Basidia suburniform, 4-spored, (12.0–)14.0–20.8(–24.0)×3.4–4.2(–5.5) μm (n=60/6), thin-walled. Basidiospores clearly thick-walled, ellipsoid to broadly ellipsoid, usually with an oil-drop, (4.0–)4.5–5.9(–7.0)×(3.3–)3.7–4.8(–5.7) μm (n=192/6), L=5.09, W=4.12, Q=1.24, cyanophilous.

#### Distribution and ecology.

Europe (Estonia, Finland, Germany, Norway, Russia – up to Ural Mts.), North America, on medium-decayed wood of angiosperms.

#### Remarks

. The type specimen of *Hyphodontianikolajevae* Parmasto reveals no essential differences from the type and other collections of *X.pruinosus* studied by us. On average, *Xylodonpruinosus* has wider basidiospores than *X.detriticus* (Table 2).

### Xylodon pumilius

Taxon classificationFungiHymenochaetalesSchizoporaceae

(Gresl. & Rajchenb.) K.H. Larss. comb. nov.

MB827075

#### Basionym.

*Hyphodontiapumilia* Gresl. & Rajchenb., Mycologia 92: 1162. 2000.

#### Type.

Argentina. Chubut. Dpto Languiñeo, Lago Engaño, on *Nothofaguspumilio*, 19 Apr 1996, A.Greslebin 650 (holotype BAFC [50031], by original designation).

For a detailed description and illustration, see Greslebin and Rajchenberg (2000). The presence of both hymenial, capitate cystidia and enclosed, tubular to moniliform cystidia with homogenous contents strongly stained by cotton blue, make this species morphologically reminiscent of *Xylodonbrevisetus* and *X.tuberculatus*. *X.pumilius* differs from both by a smooth hymenium and thick-walled basidiospores.

### Xylodon rickii

Taxon classificationFungiHymenochaetalesSchizoporaceae

(Hjortstam & Ryvarden) K.H. Larss. comb. nov.

MB827076

Figure 1

#### Basionym.

*Hypochniciumrickii* Hjortstam & Ryvarden, Mycotaxon 15: 271. 1982. ≡ *Odontiapolycystidifera* Rick, Iheringia, Sér. Bot. 5: 163. 1959. Nom. inval. (Code Art. 40.1).

#### Type.

Brazil. S. Salvador, 5 Apr 1944, Rick 20847 (holotype PACA, by original designation).

For a description, see Hjortstam and Ryvarden (1982). Gorjón (2012) could not verify the presence of large capitate cystidia, similar to those present in *X.magnifica* and included in the original description by Hjortstam and Ryvarden (1982). We restudied the isotype in herbarium O and can confirm that these large cystidia do exist, which supports a possible position of this species close to *X.detriticus* and *X.pruinosus*.

### Xylodon ussuriensis

Taxon classificationFungiHymenochaetalesSchizoporaceae

Viner sp. nov.

MB825356

Figure 12

#### Type.

RUSSIA. Primorie: Khasan Dist., Kedrovaya Pad Nat. Res., on angiosperm wood, 24 Jul 2016, I.Viner KUN 1989* (H) – ITS sequence, GenBank MH324468.

#### Etymology.

Ussuriensis (lat., adj.) – from the river Ussuri in Russian Far East and adjacent China.

#### Description.

Basidiocarps effused, up to 10 cm in longest dimension. Sterile margin white to pale ochraceous, floccose, up to 1 mm wide. Hymenial surface pale ochraceous, grandinioid to odontoid; projections rather regularly arranged, from 100 µm to 250 μm high, 90–110 μm broad at base, 6–8(–9) per mm. Hyphal structure monomitic, hyphae clamped, faintly cyanophilous, thin-walled. Subicular hyphae interwoven, (3.0–)3.4–6.2 μm in diam. (n=30/3). Tramal hyphae subparallel, subhymenial hyphae short-celled, 1.9–3.9 μm in diam. (n=30/3). Large rhomboid or stellate crystals rarely present in trama and subiculum, 10–19 μm in diam. Cystidia of three types: a) large, thin- or fairly thick-walled (wall up to 2.8 μm thick) cystidia of subicular, tramal or subhymenial origin, cylindrical, spathuliform, almost capitate or with one intercalary inflation at the upper part, (64.0–)71.0–188.9(–220.0)×(5.0–)5.7–9.4(–11.9) μm (n=30/3), often apically encrusted by large rhomboid crystals, b) astrocystidia of subhymenial origin, bearing a stellate crystalline cap 15–17×4.5–4.8 μm, sometimes rare, c) cystidia of subhymenial origin, thin-walled, varying from fusoid to cylindrical or submoniliform, rarely forked, 40.0–84.0(–92.0)×5.0–9.0(–11.4) μm (n=30/3). Basidia suburniform, 4-spored, 14.7–22.8(–24.0)×3.4–4.9 μm (n=30/3), thin-walled. Basidiospores clearly thick-walled, ellipsoid to broadly ellipsoid, usually with an oil-drop, (4.8–)5.1–6.0×3.8–4.6 μm (n=92/3), L=5.48, W=4.21, Q=1.30, cyanophilous.

**Figure 12.**
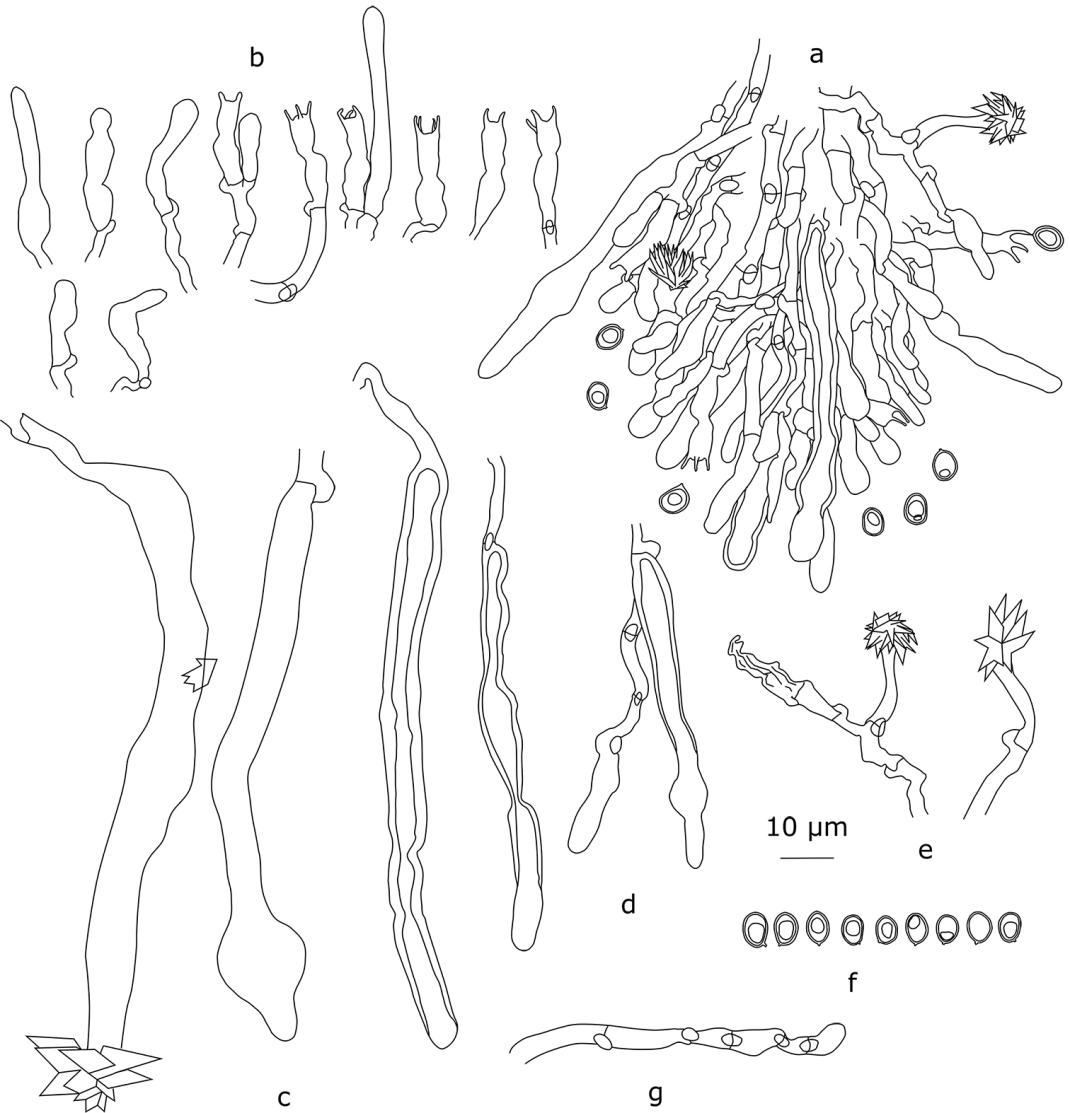
*Xylodonussuriensis* (holotype): **a** section through an aculeus **b** basidia, basidioles and hymenial cystidia **c** thick- and thin-wall tramal cystidia **d** thick- and thin-wall subhymenial cystidia **e** astrocystidia **f** basidiospores **h** short-celled hyphae from aculei.

#### Distribution and ecology.

East Asia (Russian Far East – Primorie), on decayed angiosperm wood; seemingly not rare in secondary oak-dominated forest.

#### Remarks.

The distinctly thick-walled tubular cystidia of *X.ussuriensis* make it different from other *Lagarobasidium*-like species treated here. Subhymenial astrocystidia found in *X.ussuriensis* are also present in some specimens of *X.detriticus* although they are apparently rare in the latter species.

## Discussion

Our study confirms the results from Larsson et al. (2006) and Larsson (2007) that *Peniophoradetritica* clusters with *Xylodonquercinus*, the type species of *Xylodon*. Here we also show that *Peniophorapruinosa*, the type of *Lagarobasidium*, belongs in *Xylodon* and is a sister species to *X.detriticus*. This contradicts the results published by Dueñas et al. (2009) who came to the conclusion that *Lagarobasidium* was a genus separate from *Hyphodontia* sensu lato. As support for that result, they published ITS sequences of *L.detriticum* and the new species *L.calongei* (GenBank FM876211 and FM876212, respectively). However, at least the sequence of *L.detriticum* (FM876211) seems to be based on a misidentification or contamination during the laboratory process. This sequence is 100% identical to several sequences of *Hyphodermaroseocremeum*, a species belonging in Polyporales (e.g. UNITE database UDB031922).

Blasting FM876212 against public sequence databases does not return any reliable results, which, if the sequence is correct, suggests that the species does not belong in *Xylodon*. Remaining species referred to *Lagarobasidium* and not already discussed include *L.cymosum* (D.P. Rogers & H.S. Jacks.) Jülich and *L.subdetriticum* (S.S. Rattan) J. Kaur & Dhingra. The former has been placed in *Hypochnicium* because of the thick-walled basidiospores but numerous subulate cystidia makes it a deviating element in that genus. Only access to sequence information can disclose its relationships. *Lagarobasidiumsubdetriticum* was originally described in *Hyphodontia* and should be retained in that genus also when the genus is taken in a restricted sense (Hjortstam and Ryvarden 2009).

For the phylogenetic analyses of *Hyphodontia* sensu lato, only nuclear ribosomal genes have so far been applied. All published results confirm that *Hyphodontia* sensu lato is polyphyletic and that most species can be referred to one of three clusters, viz *Hyphodontia* sensu stricto, the *Kneiffiella* cluster and the *Xylodon* cluster (including *Lyomyces*). Within these clusters the relationships are not well resolved when the ribosomal genes are the sole source for genetic information. On such detailed level, analyses become highly sensitive to sampling and outgroup choice. It is clear that both a wider sampling and more markers must be included in analyses in order to establish a stable genus level classification for all species that have been referred to *Hyphodontia* in a wide sense.

## Supplementary Material

XML Treatment for Xylodon attenuatus

XML Treatment for Xylodon crystalliger

XML Treatment for Xylodon detriticus

XML Treatment for Xylodon magnificus

XML Treatment for Xylodon nongravis

XML Treatment for Xylodon pruinosus

XML Treatment for Xylodon pumilius

XML Treatment for Xylodon rickii

XML Treatment for Xylodon ussuriensis
